# Gut Microbiota: A Modulator of Brain Plasticity and Cognitive Function in Ageing

**DOI:** 10.3390/healthcare3040898

**Published:** 2015-09-29

**Authors:** Katherine Leung, Sandrine Thuret

**Affiliations:** Institute of Psychiatry, King’s College London, The James Black Centre, 125 Coldharbour Lane, London SE5 9NU, UK; E-Mail: Katherine.Leung@kcl.ac.uk

**Keywords:** gut microbiota, inflammation, gut-brain axis, cognitive function, ageing, probiotics, adult hippocampal neurogenesis, fatty acids

## Abstract

Gut microbiota have recently been a topic of great interest in the field of microbiology, particularly their role in normal physiology and its influence on human health in disease. A large body of research has supported the presence of a pathway of communication between the gut and the brain, modulated by gut microbiota, giving rise to the term “microbiota-gut-brain” axis. It is now thought that, through this pathway, microbiota can affect behaviour and modulate brain plasticity and cognitive function in ageing. This review summarizes the evidence supporting the existence of such a connection and possible mechanisms of action whereby microbiota can influence the function of the central nervous system. Since normalisation of gut flora has been shown to prevent changes in behaviour, we further postulate on possible therapeutic targets to intervene with cognitive decline in ageing. The research poses various limitations, for example uncertainty about how this data translates to broad human populations. Nonetheless, the microbiota-gut-brain axis is an exciting field worthy of further investigation, particularly with regards to its implications on the ageing population.

## 1. Introduction

Until recently, apart from the study of infection on brain function, the fields of neuroscience and microbiology were rarely studied together. However, progress in the field of gut microbiota and its influence on human health in disease, such as in obesity and inflammatory bowel disease, has triggered an interest in the manner in which this community affects normal physiology. An increasing amount of evidence has shown that gut microbiota also play a role in the function of the central nervous system (CNS) through metabolic, neuroendocrine and immune pathways [1]. In particular, studies have illustrated an association between gut flora composition and cognitive processes such as learning and memory. Intestinal microbiota additionally contribute to the early development of normal social and cognitive behaviours [2].

Similarly fascinating, and the subject of focus of this review, is the role of the microbiota in older age with regards to its impact on brain plasticity and cognitive function. We will explore the evidence supporting the existence of a microbiota-gut-brain axis and discuss the possible underlying mechanisms by which microbiota modulates neural function, all in the context of ageing and cognitive decline. Moreover, as modulation of gut microbiota may be a novel therapeutic pathway for neurological disorders in ageing, we will evaluate possible treatment strategies such as probiotics and dietary interventions for disorders associated with aberrant signalling from the gut to the brain.

## 2. Microbiota in the Gut

The skin and mucosal surfaces of vertebrates contain an extensive array of microbiota encompassing bacteria, fungi, parasites, and viruses. In particular, more than 100 trillion bacteria reside in the human gastrointestinal (GI) tract, which is remarkably 10–100 times more than the quantity of eukaryotic cells in our bodies [3]. Colonisation of gut microbiota begins at birth and is established by the first three years of life [4]. Numerous years of co-evolution have led to a mutualistic symbiosis between host and microorganism. As such, gut microbiota contribute to various important developmental and homeostatic processes in adult life. For example, they influence metabolism by breaking down complex polysaccharides in the diet [5]. In addition, microbiota regulate gut motility, GI barrier homeostasis and fat distribution [6]. Their influence further extends to immunology through the development of gut-associated lymphoid tissues and prevention of pathogen colonisation [7]. Gut microbiota are also known to affect host energy metabolism and mitochondrial function [8].

The complex interplay of host and gut microbiota means that should this relationship be disrupted, microbiota could possibly cause or contribute to disease. Accordingly, there has been a recent focus into efforts to further delineate the diversity of these organisms, understand their role in physiology, and ultimately discover methods of preventing or treating disease through the manipulation of the microbial gut community. Within the past 10 years, progress in the field has been significantly assisted by advances in molecular and metagenomic tools, which allow for the assessment of ones microbiota composition at a genetic level [9]. Most recently, the Human Microbiome Project aimed to encourage the development of new technologies and provide a framework of metagenomic analysis through the addition of 900 reference bacterial genome sequences [10].

Using metagenomic sequencing on faecal samples, the number of microbial species present in the adult gut has been estimated to be around 1000, with each individual harbouring roughly 160 of these species [3]. Although the variety of individual microorganisms varies greatly between individuals, it has been proposed they fall into three separate enterotypes, each characterised by a single microbial genus; namely *Bacteroides*, *Prevotella* or *Ruminococcus* [11]. The composition of the gut microbial community is dynamic and affected by numerous factors including maternal transmission, GI infections, genetics, age, stress, and medication [6].

Diet in particular can exert a profound effect on the gut microbiota profile and numerous human and animal studies have illustrated distinct changes in the microbial composition of subjects consuming different diets. For instance, a comparative study on subjects on a long time Western style or rural African diet found they presented with significant differences in bacterial phenotypic profiles [12]. Furthermore, through sequencing faecal samples of 98 individuals, Wu *et al.* discovered an association between *Bacteroides* enterotypes and high animal fat or protein diets, while the *Prevotella* enterotype was associated with a high carbohydrate diet [13]. Specific carbohydrates, such as polysaccharides, fructans, and inulins, can individually exert different effects on the composition and metabolic activities of gut microbiota [14].

Infection and disease can also briefly affect natural gut flora composition and consequently have harmful effects on the host. For example, disruption of gut microbiota in young mice leads to long-term changes in visceral pain perception, characteristic of stress-related disorders such as irritable bowel syndrome (IBS) [15]. Altered gut microbial compositions have also been linked with inflammatory bowel disease and obesity [16]. Additionally, drugs, such as antibiotics, antacids, and H_2_ receptor blockers can have profound effects on gut microbiota [17,18]. All in all, it is probable that the microbiota profile is a useful reflection of the health and environmental history of an individual and may play a role in disease and treatment response.

## 3. Microbiota-Gut-Brain Axis

The connection between the CNS and GI tract is well established and essential in the modulation of gut function and homeostasis. Apart from research into the role of the gut-brain axis relating to these functions, there has been recent emphasis on how these interactions affect other aspects of human health, such as mental health. It is now known that the reciprocal communication between gut and brain involves neurological, metabolic, hormonal, and immunological signalling pathways and disturbance in these systems can result in altered behaviour [19]. For example, gut inflammation is associated with changes in gut-brain interactions and there is a high co-morbidity between inflammatory bowel disorder with anxiety [20]. The complex relationships between gut flora and the host has given rise to the notion of the microbiota-gut-brain axis [19].

Studies supporting the existence of this concept were carried out on animals maintained in a germ free environment and those with pathogenic infections. Below we summarise the key findings from each area of research in terms of the role of gut microbiota in cognitive function.

### 3.1. Germ-Free Animals

The rationale behind the experimentation on germ-free animals lies in the opportunity to assess the impact of a gut completely absent of microbiota on behaviour. The foetal gut is sterile in-utero and maintaining these animals in a sterile environment after birth means that post-natal gut colonization does not occur. It further allows for the comparison of findings against control animals with a “normal” gut microbial composition and the investigation of an individual intervention (e.g., probiotics) on the axis.

In 2004, Sudo *et al.* published the first report demonstrating that commensal gut microbiota is involved in the development of the hypothalamic-pituitary-adrenal (HPA) axis [21]. In reaction to restraint stress, germ-free mice presented with significantly higher levels of plasma corticosterone and adrenocorticotropic hormone compared to control mice with a normal gut microbial composition. The exaggerated HPA response was fully corrected with *Bifidobacterium infantis* colonization and partially corrected by colonization of faeces from the control group. However, this was only achieved when the intervention was applied at an early stage, suggesting that initial exposure to microbes is essential for the inhibitory neural regulation of the HPA axis. The HPA axis is known to be important in learning and memory, and a dysfunction in the axis can lead to impaired hippocampal memory [22].

The study further showed that germ-free mice presented with decreased levels of brain-derived neurotrophic factor (BDNF) expression in the cortex and hippocampus together with reduced expression of the NR2B subunit of the N-methyl-D-aspartate (NMDA) receptor. BDNF is a growth factor recognised for its involvement in brain plasticity and as a regulator of neural growth, survival and function [23]. As such, it is involved in the control of numerous aspects of emotional and cognitive behaviours, such hippocampal-dependent learning [24]. Similarly, NMDA receptors are involved in memory formation through the control of synaptic plasticity [25]. Since the limbic system, which is a set of brain structures including the prefrontal cortex and hippocampus, is involved in the HPA response to stress, it may be that altered cognitive processing in these areas have a role in the abnormal HPA response by germ-free mice. Additionally, Gareau *et al.* found that germ-free mice display deficits in non-spatial and working memory, accompanied with a reduction in BDNF and c-fos [26]. c-fos is an early gene product which targets cAMP response element binding protein (CREB), a protein required for hippocampus-dependent long-term memory formation [27].

Contrasting results have since been produced by other groups, such as Neufeld *et al*. [28]. Though they found a similar decrease in expression of the NMDA receptor NR2B subunit, their germ-free mice exhibited an increase in the hippocampal expression of BDNF mRNA. Reasons for these discrepancies are unclear; it may be due to different methods of mRNA expression analysis or experimentation on different genders of mice.

Other studies have noted that microbiota have an important influence on the development of cognitive processes in young mice [2]. Depletion of a normal gut microbiome in early life, especially during the post-weaning period, may affect the configuration of cognitive and social behaviours in the brain through the alteration of neuropeptides such as vasopressin and oxytocin [29,30]. In fact, there is a potential role of intestinal flora in the complex pathophysiology of autism spectrum disorder (ASD), a neurodevelopmental condition characterised by significant social and behavioural impairments [31,32]. Interestingly, treatment of an autism mouse model with probiotics have shown to ameliorate ASD-related traits [33]. Discussion of the role of microbiota in social cognition is not discussed in detail here but has been reviewed by other authors [34,35,36].

### 3.2. Bacterial Infections

Studying the impact of infection on the brain and behaviour of animals has been important in further uncovering the role of the microbiota-gut-brain axis. Bercik *et al.* investigated how chronic gut inflammation alters behaviour through infection of *Trichuris muris*, an organism closely associated to the human parasite *Trichuris trichiura* [6]. They revealed that the infected mice showed increased anxiety-like behaviour and decreased expression of BDNF. Furthermore, there was an increase in tumour necrosis factor α (TNF-α) and interferon-γ (IFN- γ), both of which are pro-inflammatory cytokines. Changes in the behaviour and cytokine levels in these mice were normalised with the anti-inflammatory agent etanercept, but this did not affect levels of BDNF expression. However, BDNF levels and behaviour were normalised with a probiotic, *Bifidobacterium longum*, but did not alter cytokine levels. The different impacts of the individual interventions illustrate varying mechanisms of action that microbiota may utilise to communicate with the brain.

Apart from studying germ-free mice, Gareau *et al.* also investigated mice inoculated with *Citrobacter* rodentium [26]. Similar findings of an increase in pro-inflammatory cytokines, TNF-α and IFN-γ, were observed in the colon. They additionally studied the effect of an enteric bacterial infection accompanied with psychological stress and illustrated that a combination of these two factors is associated with memory deficits, 30 days after infection [26]. The group hypothesized that an infection is able to prime the HPA axis, through pathways involving either serotonin or corticotrophin-releasing factor, such that subsequent stress exposure results in memory impairment. Remarkably, probiotic treatment 7 days before and during the infection normalised serum corticosterone levels and prevented changes in cognitive behaviour. It also prevented a decrease in hippocampal BDNF normally induced by *C. rodentium*.

Altogether, the data suggest that infection, inflammation, and stress can influence the CNS, particularly in behaviour and cognitive function. Increasing insights into the role of the gut-brain axis on cognitive function in humans and animals with inflammatory bowel disease and IBS is further evidence of this influence [37]. In addition, it seems that normalisation of the microbiota can prevent abnormal changes in behaviour and presents as a possible therapeutic target.

How does all this research relate to the role of gut microbiota in cognitive decline during old age? Below we explore the changes that occur in ageing in terms of the composition of the gut microbial community, inflammation, and GI function. We will then investigate how ageing and cognition are implicated through these differences.

## 4. Microbiota and Inflammation in Ageing

### 4.1. Gut Microbial Profile and GI Function

Through the expansion of studies from healthy adults to both children and the elderly, we have begun to uncover the variation in gut microbial profiles throughout ageing. It is clear that gut microbiota composition modifies as its host matures: it is relatively stable from 20 to 75 years old whereas microbial organisation in centenarians is significantly different from the adult-like pattern [38]. Though both *Bacteroidetes* and *Firmicutes* are dominant in the adult and elderly gut (95% and 93% respectively), distinct changes occur in the *Firmicutes* subgroups, with multiple members in this phylum decreasing in number [39]. There is also evidence of an increase in proportions of facultative anaerobes, such as *Staphylococcus* and *Bacillus*, and Proteobacteria such as *E. coli* in older age [40]. Several studies have reported a decrease in Bifidobacteria in the elderly [41,42,43], a strain considered to be an important probiotic with functions in maintaining gut homeostasis and preventing pathogenic infection [44].

A reason for the transformation in gut microbiota composition is the decline in GI function witnessed in old age. In the elderly, a decrease in intestinal motility affects defecation which in turn leads to constipation [45]. As a result, there is a reduction in bacterial excretion, leading to an increase in the breakdown of pancreatic enzymes which adversely alters gut function [46]. Other age-related factors such a decline in salivary function, digestion, and dentition may also affect the GI microbiome through time [47]. Additionally, age-related neurone degeneration within the enteric nervous system can alter GI motor function [48].

However, findings into the variation of gut bacteria composition through age have not been consistent. For example, differing results have been found in the numbers of *Bacteroides* between young and elderly subjects [42,43]. There is greater inter-individual variation in the composition of the GI microbiome in adults over 65 years old compared to younger adults [49]. As such, the mean quantity of a particular bacterium in the elderly disguises a significant inter-individual invariability and precisely defined conditions are required to yield consistent changes in gut microbiota. Faecal specimens used in the majority of the studies also do not offer an accurate representation of microbes in the upper GI tract, as the caecum is the main indicator of microbiome function [50].

Nonetheless, though unpredictable, it is probable that one’s gut microbial profile changes over time. It may be that old age alone is not a sufficiently strong influencing factor to alter the gut flora composition [51]. Due to the huge number of confounding factors affecting the composition of gut microbiota, it is difficult to control for these variants and elicit the influencing power of age. For these reasons, determining whether age-related changes in gut flora contribute to the parallel decline in cognitive function is challenging. A combination of events, such as antibiotic treatment or infection, may have a more significant impact on mental health in the elderly.

In fact, more than three quarters of those aged 65 or older use at least a single prescription medication, which can lead to unfavourable side effects on the GI tract [52]. For example, the use of opioids is linked to constipation [53] and the use of non-steroidal anti-inflammatory drugs with dysfunction in the GI defence system [54]. Furthermore, proton pump inhibitors, which are used to treat peptic ulcers, can trigger bacterial overgrowth of the upper GI tract through changes in the pH [17]. The use of broad-spectrum antibiotics can cause widespread disturbance in gut microbial composition and increase risk of *Clostridia difficile* infection, which in turn can reduce the biodiversity of gut microbiota [55]. As such, these medications can have implications on the microbiota-gut-brain axis and thus influence CNS function.

### 4.2. Inflammation and Immunity

Immunosenescence with chronic low-grade inflammation is characteristic in older age. This phenomenon, known as “inflammageing”, is distinguished by inflammation mediated by NF-kB and a loss of naïve CD4 T-cells [56]. A weakening of cell-mediated responses and reduction of the T cell receptor repertoire has been shown in animal models upon ageing [57]. In addition, clonal expansion of specific immune cells is impaired due to compromised cell division caused by a progressive shortening of telomeres through ageing [58]. Notably, the GI microbiota of the elderly express a pro-inflammatory phenotype, as evidenced by a reduction in Vitamin B12 synthesis and microbial reductase activity together with an increased incidence of DNA damage and immune compromise [50].

Inflammageing is thought to have a relationship with gut microbe structure and composition [59]. In 2010, Biagi *et al.* found that an increase in the pro-inflammatory cytokines IL-6 and IL-8 in centenarians correlated with enriched levels of Proteobacteria and a decrease in several butyrate producing bacteria [38]. This group presented with a significantly higher inflammation score compared to other age groups. Through immunophenotyping, they also confirmed the expected age-related T-cell changes. Taken together, their findings support that age-associated changes in the gut microbiota profile may either play a part in inflammageing or are influenced by the systemic inflammatory status. They further hypothesized that in order to attain longevity; a remodelling of gut microbiota should take place, in which equilibrium between inflammatory and anti-inflammatory processes is achieved.

Indeed, inflammation is associated with a number of age-related diseases and neurodegenerative disorders, for instance Parkinson’s Disease, Alzheimer’s disease, multiple sclerosis, and motor neurone disease [60]. Claesson *et al.* showed that gut microbial composition and inflammation is directly linked to health outcomes [49]. They analysed the microbiota composition and health outcomes in 178 patients living in different residences. Statistical analysis illustrated a clear difference in the microbial profiles depending on residence location, which significantly correlated with measures of nutrition, inflammation, frailty, and co-morbidity. A decrease in community-associated microbiota and an increase in pro-inflammatory cytokines, as detected in long-stay hospital patients, were linked to increased frailty.

## 5. Mechanisms by Which Microbiota Affect CNS Function

**Figure 1:**
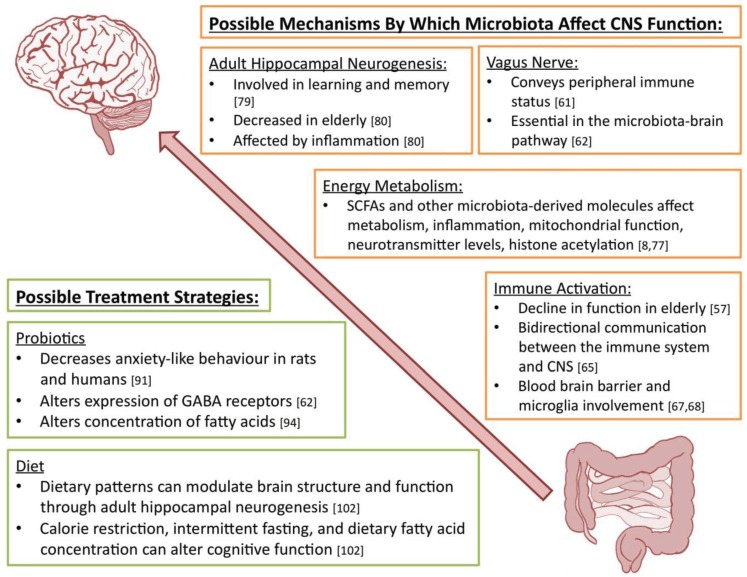
A summary of the various possible mechanisms by which microbiota can affect CNS function together with possible treatment strategies to prevent changes in behaviour and cognitive function in ageing. SCFA: short chain fatty acids; CNS: central nervous system.

We have seen a considerable amount of evidence supporting the influence of microbiota on behaviour and health. The mechanism through which this is achieved, however, is currently unclear. These are summarised in Figure 1.

One aspect that has been researched in several studies is the role of the vagus nerve. Also known as the tenth cranial nerve, it is responsible for the function of several organs, including the control of the heart rate and gut motility. The vagus nerve also conveys peripheral immune status to the CNS, for example by signalling the presence of pro-inflammatory cytokines, for instance IL-1 [61]. Vagus-dependent pathways have been shown to be involved in microbiota-brain communication, with vagotomy preventing microbiota-modulated changes in behaviour [62]. In the same study, treatment with probiotics failed to have an ameliorative effect on the behaviour of vagotomised mice, illustrating the significance of this pathway. Similar findings have since been found for other substances such as the previously mentioned probiotic, *Bifidobacterium longum* [63]*.* However, in Bercik’s study of *Trichus muris* on mice behaviour, vagotomy before infection failed to prevent anxiety-like behaviour, suggesting other vagus-independent mechanisms are in play.

Since gut microbiota can exert direct effects on the immune system, immune activation is a likely pathway in conveying the influence of bacteria on the CNS [64]. The immune system plays a crucial role in maintaining the homeostasis of the GI tract and thus the maintenance of health. As mentioned previously, there is a decline in immune function in ageing, and so microbiota-brain communication may be altered in the elderly, leading to changes in behaviour. Supporting this is the fact that behavioural changes are observed in those with systemic infections as, similar to the gut, there is reciprocal communication between the CNS and the immune system [65]. Moreover, effects of gut microbiota and probiotics on inflammatory cytokines can have a direct effect on the brain. Recent interest has been focused on the immune defence of the central nervous system, specifically microglial function, in germ-free mice [66]. Studies have found that metabolites of gut microbiota can modulate the maturation and function of microglia, thereby affecting CNS function [67]. Other groups have shown that gut microbes can also regulate the permeability of the blood brain barrier (BBB), a highly selective barrier essential in protecting the brain from potential toxins [68]. Given that the function of both the BBB and microglia may deteriorate through age, these mechanisms are particularly relevant with regards to the role of microglia in elderly cognitive decline [69,70].

Additional studied and potential mechanisms implicate host energy metabolism and are closely related to the effects of bacterial metabolites. Particular interest has been focused on short chain fatty acids (SCFAs), which is the main product of dietary fibre fermentation by gut microbes in the large intestine [71]. SCFAs regulate the metabolism of free fatty acids, glucose, and cholesterol in the body through various cell-signalling cascades involving G protein-coupled receptors [8]. They also have neuro-modulatory and epigenetic effects through histone acetylation and SCFAs has been shown to improve cognitive function in animal models for neurodevelopmental and neurodegenerative diseases [72]. However, research by MacFabe has shown administration of a specific SCFA, namely propionic acid (PPA), can induce ASD-linked behavioural traits and neurochemical changes [73]. These changes, which include neuro-inflammation, elevated oxidative stress, and antioxidant depletion, may contribute to mitochondrial dysfunction, which is common in ASD patients and is linked to other neurodegenerative diseases such as Alzheimer’s and Parkinson’s [74,75]. PPA also has effects on neurotransmitter availability, gap junction function and immune function [73]. For example, SCFAs are associated with elevated levels of phosphorylated CREB, leading to increased levels of catecholamines [76]. Other microbiota-derived molecules such as modified peptides, oligosaccharides and amino acids may similarly have a role in the microbiota-gut-brain axis, as reviewed by Donia and Fischbach [77].

Another possible pathway by which microbiota can affect the CNS is through alterations in adult hippocampal neurogenesis (AHN). It has been discovered that the adult brain has the capacity to generate new neurones in discrete areas within the hippocampus and lateral ventricles [78]. AHN is involved in learning and memory and affected in a variety of neurological disorders such as epilepsy, depression, Alzheimer’s disease, and Parkinson’s disease [79]. A decrease in adult neural stem cells and AHN is observed in the elderly with a parallel decline in cognitive function through ageing [80]. In fact, increased levels of a chemokine known as CCL11, which increases in age, has been associated with a decline in AHN and is accompanied with impaired cognitive function and synaptic plasticity [80]. Furthermore, inflammation disturbs the functional integration of neurons from the adult brain to existing neural circuits, and could be associated with cognitive decline in ageing [81]. With regards to gut microbiota, a recent study by Ogbonnaya *et al.* found altered levels of hippocampal neurogenesis in germ free mice [82]. They also found AHN was not influenced by post-weaning microbial colonisation, indicating there may be a “critical window” in which microbiota affects AHN.

Due to the involvement of similar metabolic and immune pathways, initial dysbiosis of the intestinal microbiota by diseases in childhood could possibly have long lasting effects on behaviour and cognitive function through the gut-brain axis. Apart from GI-tract associated disorders, many other chronic illnesses such obesity, asthma, allergy, and type 1 diabetes have also been linked to lifelong alterations in gut microbiota, metabolic, and immune function [83,84,85]. As many of these diseases start early in life, changes in gut flora composition may be secondary to neonatal environmental factors such as infant diet, medication, and mode of delivery. Interestingly, an increase in childhood allergies and asthma may be related to a parallel increase in caesarean sections, which have also been associated with coeliac disease and obesity [86,87,88]. Moreover, many of the pathophysiological effects of microbiota disturbance, including altered immune function and metabolic consequences such as mitochondrial dysfunction, are shared between neurodevelopmental and age-related neurodegenerative conditions. It could therefore be possible that early disruption of the microbiome may have effects that are not realised until old age. For this reason, epidemiological studies studying the gut microbiota throughout life and the long-term effects of factors disturbing its composition will be of great value.

## 6. Possible Treatment Strategies

Taken together, interventions to normalise the microbiota composition and influence age-related processes could potentially have ameliorative effects on cognitive function. Below we discuss the use of probiotics and dietary changes in the modulation of mental health, which are also summarised in Figure 1.

### 6.1. Probiotics

A probiotic is defined as a live organism that, if given in sufficient quantities, exerts beneficial effects on the host [89]. There is evidence for the use of probiotics for reducing anxiety in individuals with altered GI function, in addition to lowering systemic inflammatory cytokines and decreasing oxidative stress [90]. Messaoudi *et al.* found *Lactobacillus helveticus* combined with *Bifidobacterium longum* decreased anxiety-like behaviour in rats and humans, accompanied with a reduction in urinary free cortisol in treated humans [91]. *Lactobacillus rhamnosus* has been found to decrease anxiety-like behaviours in mice and alter expression of GABA receptors in the brain, a receptor known to play a role in anxiety disorders [62]. Probiotics have also been promising as a potential preventative strategy for depression in human trials [92]. Moreover, there is evidence that the concentration of fatty acids, namely arachidonic acid and docosahexaenoic acid, are increased in mice that received a strain of *Bifidobacterium breve* [93]. It is known that both fatty acids contribute to several neurodevelopmental processes, including neurogenesis and neurotransmission, and can impact cognitive functions such as learning and memory [94].

A recent study investigated the effect of VSL#3, a probiotic mixture previously shown to reduce visceral pain hypersensitivity on IBS models, on long-term potentiation (LTP) [95,96]. LTP is a marker of brain plasticity and is reduced in the hippocampus of middle and old aged rats. The group hypothesised that, since the GI microbiome alters with age, aged-related changes in LTP could be attenuated with VSL#3. Remarkably, VSL#3 lead to significant changes in the gut microbiota composition and increased brain tissue expression of genes associated with inflammation and neural plasticity, such as BDNF and synapsin. In another study, Davari *et al.* found that probiotic administration in rats with metabolic changes in learning and memory, secondary to diabetes mellitus, reversed a decline in synaptic activity, and additionally restored a disturbance in hippocampal LTP [97]. Recent data further show that certain bacterial species, specifically Bifidobacteria strains, have a positive impact on cognitive processes [98].

Though there is a fair amount of evidence supporting the effectiveness of probiotics, the mechanism of action of these organisms is less clear. They may competitively exclude gut pathogens through the formation of a physical barrier, preventing entry, or production of antimicrobial properties [99]. Alternatively, they may stimulate the immune system by stimulating the production of anti-inflammatory cytokines [89]. As mentioned previously, they may also communicate with the CNS through the vagus nerve [62]. Nonetheless, these studies provide confirmation of the presence of a microbiota-gut-brain axis, and it is clear that probiotics can modulate aspects of brain function. Further research will hopefully identify additional organisms and uncover their underlying mechanism of action.

### 6.2. Diet

Several epidemiological studies in elderly subjects have found links between diet and cognitive function [100]. Similar diet-related changes in cognitive flexibility have been found in mice fed either a high sucrose or high fat diet, possibly secondary to a shift in gut microbiota composition [101]. Recently, a large body of research has further suggested that dietary patterns can modulate brain structure and function throughout life through AHN [102]. Calorie restriction, intermittent fasting, and protein content in the diet have all been shown to exert effects on AHN. For example, maintaining rodents on a calorie-restricted diet can prevent age-related decline in cognitive function, possibly through the maintenance of NMDA receptors in the hippocampus, which normally decline with age [103]. Witte *et al.* conducted the first study displaying the positive effects of calorie restriction on memory performance in an elderly cohort [104]. Similarly, intermittent fasting in mice resulted in an increase in markers of brain plasticity [105].

Since fatty acids seem to play an important role in shaping the gut microbiota metabolism, it is unsurprising that they have been considered as a dietary means to impede cognitive decline in ageing. Human and animal studies on omega-3 polyunsaturated acids (n-3 PUFA) have shed light on their neuro-protective roles through pathways of synaptic plasticity, neuro-inflammation, and oxidative stress [106]. Supplementation of n-3 PUFA has been shown to increase hippocampal neurogenesis and the fatty acid docosapentaenoic acid has shown to modulate synaptic plasticity in aged rats [107,108]. As such, many clinical trials have explored the role of fatty acids on neurodegenerative disorders such as Alzheimer’s, although the data has not been consistent [109]. L-carnitine, which transports fatty acids into mitochondria for oxidation, has also been shown to have neuro-protective effects, possibly through enhancing neurotransmission and mitochondrial function [110,111]. As L-carnitine is metabolised by gut microbiota, these effects may be achieved through the microbiota-gut-brain axis, and is worthy of further research as a potential therapeutic agent in the elderly.

## 7. Conclusions

We have seen an array of evidence supporting the notion that microbiota can modulate gut-brain interactions and further influence immune markers and markers of brain plasticity. In addition, chronic inflammation and a transformation in gut microbiota through age parallels a decline in cognitive function. As such, the development of therapeutic strategies for diseases characterised by cognitive decline through alteration of the microbiota-gut-brain axis is an appealing possibility, particularly in the context of a global ageing population. However, much work still remains before this becomes a reality. For example, translation of animal data onto human models and additional research to recognise the numerous components involved in the microbiota-gut-brain axis is required. Of note, the human microbiota-gut-brain axis significantly differs from the rodent axis due to differences in brain structure, particularly in the prefrontal cortex and frontoinsular regions. In addition, many of the animal studies focusing on the effects of gut microbiota have been centred on neurodevelopment, not ageing. Through this review we have illustrated that the latter is a subject certainly worthy of further research.

In terms of the potential treatment strategies, there is currently very limited research of the long-term effects of many of these agents, for example probiotics, in human populations. Investigation of probiotics is further hindered by the varying quality control and efficacy of such a large family of compounds. It is also important to note that achieving a risk reduction in a certain disorder may concurrently increase the risk for another, as many of the disorders involving the microbiota-gut-brain axis have diverse pathophysiological mechanisms and risk factors. Another point to consider is that of the dose and timing of probiotic and dietary interventions, as illustrated by the effects propionic acid at varying doses.

Nonetheless, the field has come a long way in the past decade and substantial developments in science and technology will hopefully bring us closer to a therapeutic pathway in the future. Carefully designed longitudinal clinical studies studying the microbial profiles of phenotyped patients throughout life, and the long-term effects of factors disturbing its composition (e.g., via probiotics, antibiotics, dietary interventions) will be of great value to expand our knowledge of a promising area of research. Of interest would be the consequences of recently introduced factors such as modern agricultural practices and antibiotics on age-related diseases [112]. Current studies in developing countries and Norway investigating infant gut microbiota colonisation and subsequent health will hopefully lead to further investigations into this complex field [113,114].
